# Adipose tissue depots and blood pressure regulation: mechanisms, biomarkers, and targets beyond obesity

**DOI:** 10.1093/ajh/hpag065

**Published:** 2026-06-20

**Authors:** Stephanie Rodriguez, Vijayvardhan Kamalumpundi, Joshua Peterson, Cally Tucker, Linhai Cheng, Elham Shams, Erin Meyers, Sefi Jawadi, Renata O Pereira, Marcelo Lima de Gusmão Correia

**Affiliations:** Department of Internal Medicine, University of Minnesota, Minneapolis, MN, United States; Department of Internal Medicine, Mayo Clinic, Rochester, MN, United States; College of Liberal Arts and Sciences, University of Iowa, Iowa City, IA, United States; College of Liberal Arts and Sciences, University of Iowa, Iowa City, IA, United States; College of Liberal Arts and Sciences, University of Iowa, Iowa City, IA, United States; Division of Endocrinology and Metabolism, University of Iowa Health Care, Iowa City, IA, United States; Division of Endocrinology and Metabolism, University of Iowa Health Care, Iowa City, IA, United States; Division of Endocrinology and Metabolism, University of Iowa Health Care, Iowa City, IA, United States; Division of Endocrinology and Metabolism, University of Iowa Health Care, Iowa City, IA, United States; Fraternal Order of Eagles Diabetes Research Center, University of Iowa, Iowa City, IA, United States; Division of Endocrinology and Metabolism, University of Iowa Health Care, Iowa City, IA, United States; Fraternal Order of Eagles Diabetes Research Center, University of Iowa, Iowa City, IA, United States

**Keywords:** blood pressure, adipose tissue, adipokine, batokine, exosome, microRNA, hypertension

## Abstract

**Background:**

Adipose tissue influences blood pressure regulation through depot-specific endocrine, inflammatory, and exosome-dependent mechanisms. While obesity is a well-established risk factor for hypertension, emerging evidence suggests that distinct fat depots may contribute to vascular dysfunction independent of total adiposity. Methods: This review synthesizes post-2020 literature on 6 anatomically defined depots: visceral, perivascular, epicardial, bone marrow, brown, and beige adipose tissue, with an emphasis on their mechanistic roles, imaging biomarkers, and translational relevance.

**Results:**

Alterations in adipose depot function can impair nitric oxide signaling, promote oxidative stress, and modulate sympathetic tone, even in the absence of obesity. Exosomal microRNAs from dysfunctional adipose tissue further disrupt vascular homeostasis. Although clinical tools for assessing depot dysfunction are limited, advances in imaging and molecular profiling may enable targeted therapeutic strategies.

**Conclusions:**

Reframing adipose tissue as a modular regulator of blood pressure, rather than a passive consequence of obesity, may unlock new venues for precision management of hypertension.

## Graphical abstract

**Graphical Abstract:**
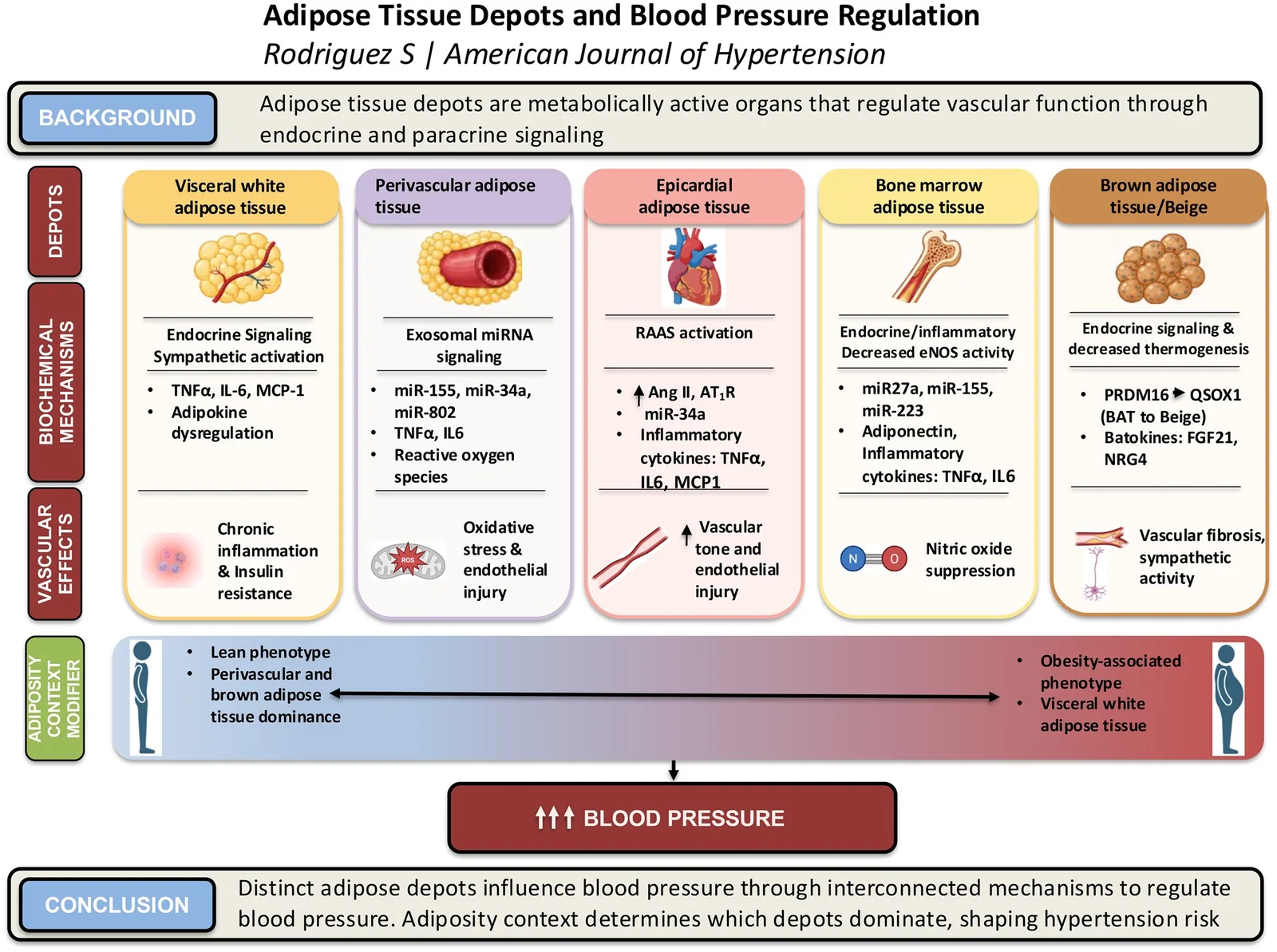

## Introduction

Adipose tissue contributes to blood pressure (BP) regulation through endocrine, inflammatory, neural, and exosomal mechanisms. Although obesity is a major risk factor for hypertension (HTN), evidence also indicates that specific adipose depots can directly influence vascular tone, endothelial function, and sympathetic activation, independent of total adiposity.^1–4^

Epidemiologic data confirm that HTN frequently develops in lean individuals. In U.S. NHANES cohorts, ∼15%-20% of adults with hypertension have a body mass index (BMI) within the normal range.^5–7^ Similar patterns are observed in Asian populations, where visceral adiposity and metabolic risk emerge at lower BMI thresholds.^6^ These findings reinforce that adipose tissue dysfunction, rather than total adiposity, may drive elevated blood pressure in a subset of individuals.

These effects are mediated by localized secretion of cytokines, adipokines, and microRNAs (miRNAs) as well as depot-specific interactions with adjacent vascular or cardiac structures.^2–4^ Depot-level studies reveal that visceral fat activates the renin-angiotensin-aldosterone system (RAAS) and promotes oxidative stress. While perivascular fat modulates vascular contractility through nitric oxide (NO) and angiotensin signaling, epicardial fat contributes to coronary inflammation and myocardial stiffening. Additionally, bone marrow adipocytes influence hematopoietic output and systemic immune tone, whereas brown adipose tissue lowers vascular resistance via thermogenic and batokine pathways. Although these pathways have been identified, their downstream mechanisms remain poorly defined in individuals living with hypertension without obesity. This knowledge gap reinforces the need to reframe adipose dysfunction as a potential driver of hypertension beyond obesity.^2^^,^^3^^,^^8^

This review focuses on the direct role of anatomically distinct adipose depots in BP regulation, independent of obesity. It synthesizes recent advances in depot-specific mechanisms, imaging biomarkers, and therapeutic strategies across 6 compartments: visceral white adipose tissue (vWAT), subcutaneous WAT (scWAT), perivascular adipose tissue (PVAT), epicardial adipose tissue (EAT), bone marrow adipose tissue (BMAT), and beige/brown adipose tissue (beige/BAT). While the review primarily emphasizes mechanistic and translational studies published from 2020 onward, several earlier foundational studies were included to provide physiologic and historical context. Defining how these depots contribute to vascular dysfunction beyond total adiposity is essential for refining HTN pathophysiology and developing more targeted therapies for individuals with hypertension with or without obesity. The major adipose depot-specific pathways linking adipose dysfunction to vascular dysfunction and elevated blood pressure are summarized in the graphical abstract.

## Cellular heterogeneity and temporal dynamics in adipose depots

Adipose depots are increasingly recognized as highly dynamic, plastic, and heterogeneous tissues rather than static lipid storage reservoirs.^9^^,^^10^ In addition to mature adipocytes, these depots contain diverse populations of immune cells, endothelial cells, fibroblasts, mesenchymal stromal cells, and adipocyte precursor populations that remodel in response to metabolic, nutritional, inflammatory, and environmental stressors.^9–11^ Recent single-cell and single-nucleus transcriptomic studies have demonstrated substantial depot-specific differences in cellular composition and functional state, particularly between visceral white adipose tissue and subcutaneous white adipose tissue, including variation in immune cell populations, endothelial populations, adipocyte progenitor states, and mesothelial-like cell populations.^9^^,^^11^ Emerging evidence further suggests that vascular disease-associated stressors alter perivascular adipose tissue composition and signaling, supporting a role for adipose tissue remodeling in endothelial dysfunction, vascular inflammation, oxidative stress, and blood pressure regulation.^9^^,^^12^ These dynamic shifts may alter local secretion of adipokines, cytokines, chemokines, and vasoactive mediators involved in vascular dysfunction.^9^^,^^10^^,^^12^ Recognition of this evolving cellular complexity supports the concept that adipose tissue functions as an active and adaptable endocrine and paracrine organ with context-specific contributions to cardiometabolic and vascular regulation.^9–12^

## Mechanisms associated with visceral white adipose tissue and blood pressure regulation

Visceral WAT regulates BP through endocrine, inflammatory, and paracrine mechanisms that operate independently of total adiposity.^1^ Compared to subcutaneous fat, vWAT is more vascularized, innervated, and immunologically active,^1^ including in individuals with normal BMI.^13^ While numerous mechanistic insights have been gained from obesity-focused research, evidence in individuals with hypertension in the absence of obesity remains limited and highlights a critical gap in the field.^14^

Visceral WAT is anatomically heterogeneous, comprising mesenteric, omental, and perirenal depots with differing inflammatory and endocrine activity.^1^^,^^15^ Perirenal fat contributes to renal sympathetic activity and local RAAS signaling,^1^ while omental vWAT is more vascularized and prone to immune infiltration.^15^

Visceral WAT secretes inflammatory cytokines such as TNF-α and IL-6, which impair endothelial vasodilation by suppressing NO and increasing oxidative stress. Although well-characterized in obesity, their role in lean individuals with hypertension remains unclear.^1^ Resistin promotes vascular contraction and RAAS activation via TLR4 in non-obese murine models, but human validation is lacking.^1^^,^^14^ Leptin, a regulator of sympathetic outflow, is typically elevated in obesity and has limited supporting data in normo-leptinemic humans with hypertension.^1^ In contrast, omentin-1, consistently downregulated in vWAT across BMI groups, exerts vaso-protective effects via AMPK-Akt signaling and is restored by tetrahydroxystilbene glycoside in hypertensive rats.^16^

Data also suggest that gut-derived metabolites may influence vWAT-mediated BP control. The short-chain fatty acid butyrate reduces local inflammation, restores TRPV4-calcium signaling, and suppresses RAAS activity. In lean hypertensive mice, oral butyrate normalized BP without affecting adiposity, highlighting a rare obesity-independent mechanism linking vWAT dysfunction to hypertension.^8^

Visceral WAT-derived exosomes modulate vascular tone through miRNAs that influence endothelial and smooth muscle function. For example, miR-155, enriched in macrophage-derived vesicles, suppresses PPARγ, reduces NO availability, and disrupts insulin signaling.^17^ In addition, miR-802-5p, which is secreted by hypertrophic adipocytes, impairs mitochondrial HSP60, increasing oxidative stress and reducing vasodilation.^17^ Similarly, miR-34a promotes macrophage polarization and elevates TNF-α and IL-6 expression, amplifying inflammation.^17^

Although these miRNAs contribute to vascular dysfunction in animal models, they remain unvalidated in human hypertension.^14^^,^^17^ Protective miRNAs such as miR-126, which enhance eNOS activity and endothelial regeneration, are reduced under metabolic stress but have yet to be studied in normal-weight individuals with hypertension.^17^

## Imaging, biomarkers, and therapeutic strategies related to visceral white adipose tissue

Quantitative biomarkers such as the Metabolic Score for Visceral Fat (METS-VF) and Visceral Adiposity Index (VAI) predict hypertension independent of BMI, supporting a direct non-BMI dependent role for vWAT in BP regulation.^13^ Registry data show that visceral fat area and visceral-to-subcutaneous fat ratio predict incident HTN across body weight categories.^14^ VAI also correlates nonlinearly with systolic and diastolic BP.^18^ However, most studies include individuals with borderline overweight, limiting conclusions about lean populations.

Targeting vWAT function, rather than reducing fat mass, offers translational potential. In diabetic mice, FGF21 gene therapy delivered to vWAT suppressed TNF-α and CCL2, increased adiponectin, and improved vascular function without affecting body weight.^18^ SGLT2 inhibitors and GLP-1 receptor agonists reduce vWAT and lower BP in humans, but it remains unclear whether these effects are adipose depot-specific or weight-mediated.^1^ To date, no trials have evaluated vWAT-targeted therapies in individuals with hypertension.

## Mechanisms associated with perivascular adipose tissue and blood pressure regulation

Perivascular adipose tissue lies adjacent to the blood vessels, allowing direct paracrine and exosomal modulation of vascular tone. Unlike other adipose depots, PVAT arises from a smooth muscle-like mesenchymal lineage and is anatomically variable.^19^

In rodents, thoracic PVAT resembles thermogenic brown fat, while abdominal PVAT is proinflammatory. Human studies show that internal thoracic PVAT retains anticontractile properties under healthy conditions but becomes fibrotic in metabolic disease.^3^^,^^19^ Proposed mechanisms underlying these preserved anticontractile effects include adiponectin-mediated vasorelaxation, enhanced NO bioavailability, activation of vascular smooth muscle potassium channels, and lower local oxidative and inflammatory signaling under physiologic conditions.^3^^,^^19^

Under physiological conditions, PVAT releases adiponectin, NO, angiotensin-(1-7), and hydrogen sulfide to support vasodilation.^3^ In hypertension, PVAT becomes inflamed, reducing NO bioavailability and increasing oxidative stress.^3^ PVAT dysfunction has been confirmed in spontaneously hypertensive rats, an obesity-independent model, where TNF-α and IL-6 are elevated.^3^

PVAT-derived exosomes influence endothelial and smooth muscle cell behavior. miR-132-3p, miR-212-3p, and miR-221-3p disrupt endothelial junctions, suppress eNOS, and promote vascular remodeling.^20^^,^^21^ Although not yet validated in lean humans with hypertension, these miRNAs are plausible mediators of obesity-independent vascular dysfunction.

## Imaging, biomarkers, and therapeutic strategies related to perivascular adipose tissue

Although no circulating biomarkers currently correlate directly with PVAT function, advanced imaging techniques are emerging. The fat attenuation index (FAI) from coronary CT reflects PVAT inflammation and predicts cardiovascular events, whereas 18F-FDG PET/CT can visualize active PVAT but lacks specificity.^22^

Notably, β3-adrenergic agonists and cold exposure improve PVAT function in preclinical models. However, mirabegron, a β3-adrenergic agonist, causes modest increases in diastolic BP that may limit their use in patients with hypertension.^19^^,^^23^ Adipose depot-targeted FGF21 therapy also reduces inflammation and enhances NO signaling, but clinical trials in the setting of HTN are lacking.^23^

## Mechanisms associated with epicardial adipose tissue and blood pressure regulation

Epicardial adipose tissue is a visceral fat depot located between the myocardium and visceral pericardium. It shares the coronary circulation and lacks a fascial barrier, enabling direct signaling to adjacent vasculature and myocardium. This anatomic and paracrine proximity allows EAT to influence coronary artery tone, myocardial stiffness, and systemic BP.^23^^,^^24^

Under physiological conditions, EAT produces adiponectin and omentin-1, which promote vasodilation and reduce oxidative stress. Dysfunctional EAT expresses increased TNF-α, IL-6, MCP-1, and angiotensin II, which activate STAT3 and NF-κB pathways, suppress NO, and increase vascular resistance.^24^^,^^25^ Dysfunction of EAT also contributes to hypertension in the absence of obesity. In adolescents with hypertension^24^ and adults with heart failure with preserved ejection fraction, EAT thickness independently predicted BP and ventricular stiffness after adjusting for BMI.^23^

In hypertensive and failing hearts, EAT-derived exosomes show enrichment of miR-34a, which promotes myocardial fibrosis through TGF-β1 activation, and miR-155, which impairs endothelial function by targeting eNOS and promoting oxidative stress.^20^ While human data in lean cohorts are lacking, these miRNAs offer potential as biomarkers and therapeutic targets.

## Imaging, biomarkers, and therapeutic strategies related to epicardial adipose tissue

The thickness and radiodensity of EAT can be quantified using echocardiography and CT.^22^ Low radiodensity correlates with inflammation and lipid content. In normal-weight individuals, elevated EAT thickness with low radiodensity may reflect depot dysfunction even in the absence of excess fat mass.^26^

The volume and inflammatory status of EAT are modifiable through interventions in humans. For instance, caloric restriction and aerobic exercise reduce EAT thickness and improve adipokine profiles, primarily in individuals with overweight.^23^ GLP-1 receptor agonists and SGLT2 inhibitors also lower EAT volume and inflammation, though their impact in normal-weight individuals with hypertension remains untested.^23^

## Mechanisms associated with bone marrow adipose tissue and blood pressure regulation

Bone marrow adipose tissue is a metabolically active adipose depot residing in long bones and vertebrae.^4^^,^^27^ It exists as constitutive BMAT, which is stable, and regulated BMAT that is responsive to inflammation, glucocorticoids, and catabolic stress. BMAT secretes adiponectin, TNF-α, and IL-6, influencing vascular tone and immune regulation.^28^

In hypertensive animals, BMAT expansion increases TNF-α and IL-6 expression, suppressing eNOS and promoting vascular smooth muscle contraction. It also promotes differentiation of proinflammatory myeloid cells, contributing to systemic vascular inflammation. These effects are particularly relevant in normal-weight individuals with osteopenia or chronic inflammation, where BMAT is often expanded despite low total adiposity.^4^^,^^27^

Exosomes derived from BMAT contain miR-27a, miR-155, and miR-223, which regulate PPARγ, eNOS, and inflammatory cell differentiation. Although linked to hypertension in preclinical models, BMAT-specific exosomes in humans remain undefined.^17^

## Imaging, biomarkers, and therapeutic strategies related to bone marrow adipose tissue

Bone marrow adipose tissue can be quantified using PDFF-MRI or 18F-FDG PET/CT. Elevated BMAT has been associated with inflammation and reduced bone density in normal-weight post-menopausal women, though no validated circulating biomarkers exist.^4^

Targeting BMAT function, rather than reducing its volume, may help mitigate vascular dysfunction. In animal models, PPARγ agonists increase BMAT mass, and enhance function by elevating adiponectin and by suppressing inflammation.^27^ Exercise and mechanical loading reduce BMAT in weight-bearing bones and improve bone-vascular signaling, though BP effects in normal-weight individuals remain unstudied. Future strategies may involve modulating BMAT-derived exosomal miRNAs or inhibiting depot-specific cytokines to restore endothelial function and lower vascular resistance.^17^

## Mechanisms associated with beige and brown adipose tissue and blood pressure regulation

Beige/BAT are thermogenic depots that dissipate energy via UCP1-mediated mitochondrial uncoupling.^22^ Classical BAT originates from Myf5+ precursors and resides in supraclavicular and perirenal regions, while beige adipocytes emerge within white fat depots in response to cold, β3-agonists, and certain diets.^29^ Unlike WAT, BAT activity does not correlate with BMI and may be reduced in normal-weight individuals with vascular dysfunction.^22^

Brown adipose tissue regulates BP through both metabolic and direct vascular effects. It secretes batokines, such as FGF21, NRG4, and IL-6 that reduce arterial stiffness, sympathetic tone, and RAAS signaling. In normal-weight hypertensive mice, BAT activation improved NO bioavailability, suppressed angiotensin II type 1 receptor signaling, and reduced systemic BP.^8^^,^^30^

Importantly, Koenen et al^31^ examined mouse beige fat to understand how it communicates with blood vessels. Loss of beige fat identity in adipocyte‑specific Prdm16 knockout mice caused abnormal perivascular fat remodeling, heightened vascular reactivity, and elevated blood pressure. Circulating enzyme QSOX1 is upregulated in Prdm16-deficient adipocytes. Deleting Qsox1 reversed vascular fibrosis and normalized vessel function, showing that beige adipocytes regulate blood pressure via QSOX1‑mediated signaling.

Furthermore, BAT-derived extracellular vesicles contain miRNAs with vasculo-protective functions, including miR-126, which promotes VEGF-mediated angiogenesis, miR-92a that impairs endothelial regeneration, and miR-210,^20^ a mitochondrial function regulatory factor under hypoxia. Although identified in rodent models, BAT-specific exosomal profiles in lean humans with HTN remain uncharacterized.

## Imaging, biomarkers, and therapeutic strategies related to brown and beige adipose tissue

18F-FDG PET/CT quantifies BAT activity and correlates with lower BP and improved vascular function, independent of adiposity.^22^ Emerging imaging techniques, such as MRI fat fraction mapping, may further differentiate active versus inactive BAT.^22^ Circulating batokines such as FGF21 and NRG4 may serve as biomarkers of BAT function, although they lack adipose depot specificity.^30^

Several novel strategies target BAT activation and WAT browning through depot-specific pathways. Dapagliflozin, an SGLT2 inhibitor, promotes thermogenic gene expression and mitochondrial biogenesis via FGFR1-LKB1-AMPK signaling cascade, enhancing UCP1 expression in both WAT and BAT.^29^ β3-adrenergic agonists such as mirabegron activate BAT and improve vascular tone and glucose metabolism, though BP effects vary by dose and sympathetic tone.^30^ Cold exposure, exercise, and polyphenol-rich diets promote beige adipogenesis and vascular health but have not been studied in normal-weight hypertensive populations.^8^ Furthermore, FGF21 gene therapy, while primarily tested in visceral fat, also enhances BAT activation, vasodilation, and endothelial function^18^

## Subcutaneous white adipose tissue

Subcutaneous WAT, the largest fat depot in the human body, is generally associated with favorable metabolic and vascular profiles. In general, scWAT exhibits lower inflammatory activity and higher adiponectin secretion than vWAT.^1^ While some studies suggest regional differences in inflammatory status, particularly between gluteo-femoral and abdominal depots, direct links to lean individuals with hypertension remain unestablished.^32^^,^^33^ Exosomes and miRNAs derived from scWAT, such as miR-23b and miR-27a, impair vasodilation in obesity but are not elevated in lean individuals.^20^^,^^34^

A summary of shared and adipose depot-specific mechanistic pathways contributing to blood pressure regulation is presented in Table 1. Detailed translational and biomarker information is provided in Table S1. Comparative imaging and biomarker features across adipose depots, including their diagnostic and prognostic relevance in hypertension, are summarized in Table 2. The hierarchy of current and emerging therapeutic strategies targeting adipose-vascular dysfunction is outlined in Table 3, ranging from clinically validated metabolic interventions to experimental adipose depot-specific approaches.

**Table 1:** Cross-depot mechanistic pathways in adipose tissue-mediated blood pressure regulation.

Mechanistic axis	Shared across depots	Depot-specific features	Species/model evidence
**Endocrine/cytokine**	↑ TNF-α, ↑ IL-6 → ↓ NO, ↑ oxidative stress, ↑ RAAS activation	vWAT: ↑ Resistin, ↓ Omentin-1 EAT: ↑ MCP-1, Ang II → STAT3/NF-κB BMAT: ↑ TNF-α/IL-6 secretion	Human + murine
**Sympathetic/adipokine**	Leptin and adiponectin regulate sympathetic tone and endothelial function	vWAT: hyperleptinemia (↑ SNS) BAT: FGF21/adiponectin axis → ↓ SNS, ↓ RAAS	Human + murine
**Oxidative stress/NO signaling**	↓ eNOS and NO bioavailability across depots	PVAT: impaired NO diffusion EAT: ROS-driven endothelial dysfunction	Primarily murine with supportive human mechanistic studies
**RAAS interaction**	Ang II upregulation; depot inflammation enhances angiotensinogen expression	vWAT and perirenal fat: activates local RAAS EAT: expresses Ang II + AT1R → fibrosis	Human + murine
**Exosomal miRNA signaling**	miR-155, miR-34a, miR-27a → ↓ PPARγ, ↓ eNOS, ↑ inflammation	vWAT: miR-802-5p PVAT: miR-132-3p/221-3p BMAT: miR-223	Predominantly murine/preclinical
**Thermogenic/metabolic**	BAT and beige depots improve vascular tone via UCP1-FGF21 axis and through QSOX1 signaling	BAT: ↑ FGF21, NRG4, IL-6 → ↓ RAAS, ↓ stiffness	Murine with emerging human evidence

**Table 2:** Biomarkers and imaging modalities by adipose depot.

Depot	Biomarkers/imaging metrics	Findings in hypertension	Clinical relevance
**vWAT**	METS-VF, VAT: SAT ratio, VAI	Higher scores predict HTN independent of BMI	Visceral obesity index correlates with metabolic HTN risk
**PVAT**	Fat Attenuation Index (FAI), 18F-FDG PET	Increased inflammation linked to endothelial dysfunction	Marker of vascular inflammation
**EAT**	CT thickness, radiodensity, EAT volume	Low radiodensity = higher inflammation and stiffness	Predictive for coronary disease and HFpEF
**BMAT**	MRI PDFF, 18F-FDG PET	Expansion associated with inflammation and low bone density	Potential immune-metabolic biomarker in lean HTN
**BAT**	18F-FDG PET/CT, MRI fat fraction, circulating FGF21	Active BAT correlates with lower BP and better metabolic profile	Target for metabolic and vascular therapies

**Table 3:** Hierarchy of therapeutic strategies targeting adipose-vascular dysfunction.

Category	Representative interventions	Mechanistic targets	Clinical readiness/limitations
**Clinically validated**	SGLT2 inhibitors, GLP-1RAs, caloric restriction ± exercise	↓ vWAT and EAT inflammation, ↑ adiponectin, improved vascular compliance	Proven metabolic and BP benefits; depot-level data limited
**Experimental/translational**	FGF21 gene therapy, depot anti-inflammatory or exosome modulation, β3-adrenergic agonists	↑ NO signaling, ↓ TNF-α, ↑ thermogenesis	Early-phase/preclinical; safety monitoring ongoing
**Speculative/emerging**	BAT transplantation, cold exposure, polyphenols, miRNA therapies	Stimulate thermogenesis, alter exosomal crosstalk	Conceptual; long-term human efficacy unknown

Although GLP-1 receptor agonists and SGLT2 inhibitors demonstrate favorable metabolic, vascular, and antihypertensive effects, distinguishing depot-specific mechanisms from generalized weight reduction remains challenging.^35^^,^^36^ Current evidence supports effects on visceral adiposity, adipokine profiles, vascular function, and inflammatory markers^37^^,^^38^; however, many observed improvements in blood pressure and vascular function likely reflect integrated systemic metabolic effects rather than isolated modulation of a single adipose depot.^36^^,^^38^

## Conclusions

Adipose tissue regulates blood pressure through depot-specific endocrine, inflammatory, and exosomal mechanisms. This narrative review underscores that visceral, perivascular, epicardial, bone marrow, and brown/beige fat each contribute to vascular dysfunction via arterial stiffness, endothelial impairment, and altered sympathetic tone.^2–4^^,^^8^^,^^14^^,^^22^

While many mechanistic studies focused on obesity, evidence implicates adipose dysfunction in BP elevation among lean individuals. For instance, vWAT inflammation, EAT thickness, PVAT-derived oxidative stress, and BMAT expansion have all been linked to hypertension independent of BMI. Beige/BAT activation also improves vascular tone in normal-weight models, further supporting adipose depot-specific contributions beyond total fat mass.

These findings call for a shift from BMI-centric paradigms toward models that account for regional adipose signaling. Advancing this field will require integration of static and functional imaging, molecular markers, and exosomal profiling to identify and target dysfunctional adipose depots aiming at improving blood pressure control. Such approaches may enable more precise, adipose depot-focused strategies for hypertension management across different body compositions and phenotypes.

## Supplementary Material

hpag065_Supplementary_Data

## Data Availability

No new data were generated or analyzed in support of this review.
