# Supplementary material for: Adipose tissue depots and blood pressure regulation: mechanisms, biomarkers, and targets beyond obesity
Source: Am J Hypertens. 2026 Jun 20;39(10):1165–71. doi: 10.1093/ajh/hpag065 (PMC13579044; doi:10.1093/ajh/hpag065)
Supplement: Supplementary file 1 [file hpag065_supplementary_data.docx]

**Supplemental Table S1.** Summary of Mechanistic, Biomarker, and Therapeutic Insights by Adipose Depot

| Adipose Depot | Key Mechanistic Contributions to BP Regulation | Representative Biomarkers / Imaging Findings | Emerging or Potential Therapeutic Targets |
| --- | --- | --- | --- |
| Visceral White Adipose Tissue | ↑ TNF-α, IL-6, Resistin → ↓ NO, ↑ RAAS, oxidative stress; ↓ Omentin-1 (vasoprotective); Gut-derived butyrate reduces inflammation and BP independent of adiposity. | METS-VF, VAI, VAT:SAT ratio; vWAT-derived miR-155, miR-802-5p, miR-34a. | FGF21 gene therapy, GLP-1RAs, SGLT2 inhibitors, butyrate supplementation. |
| Subcutaneous White Adipose Tissue | Generally vasoprotective; ↑ Adiponectin, ↓ Inflammatory tone vs vWAT; No strong mechanistic link to HTN in lean subjects. | None validated; miR-23b, miR-27a identified in obesity but not lean HTN. | Not established; potential role of regional (gluteofemoral) preservation. |
| Perivascular Adipose Tissue | Normal: Adiponectin, NO, Ang-(1–7), H₂S support vasodilation. In HTN: ↑ TNF-α/IL-6 → oxidative stress, endothelial dysfunction. | Fat Attenuation Index (FAI) via CT; 18F-FDG PET uptake; PVAT miR-132-3p, miR-212-3p, miR-221-3p. | Depot-targeted FGF21 therapy; β3-agonists (limited by BP effects); anti-inflammatory modulation. |
| Epicardial Adipose Tissue | ↑ TNF-α, IL-6, MCP-1, Ang II → activate STAT3/NF-κB, suppress NO, ↑ vascular stiffness; ↓ Adiponectin, Omentin-1. | Echocardiographic or CT EAT thickness/radiodensity; EAT miR-34a, miR-155. | Caloric restriction, exercise, GLP-1RAs, SGLT2 inhibitors, exosomal miRNA modulation. |
| Bone Marrow Adipose Tissue | ↑ TNF-α, IL-6 → ↓ eNOS, ↑ vascular contraction; promotes proinflammatory myeloid differentiation. | PDFF-MRI, 18F-FDG PET; BMAT miR-27a, miR-155, miR-223. | PPARγ agonists, exercise/mechanical loading, targeting BMAT exosomal signaling. |
| Brown / Beige Adipose Tissue | ↑ Batokines (FGF21, NRG4, IL-6) → ↓ RAAS, ↓ sympathetic tone, ↑ NO; thermogenic activation lowers BP.  Loss of beige identity (Prdm16 ablation) → ↑ QSOX1 secretion, ↑ ANGII-mediated vascular contraction, ↑ fibrosis, ↑ BP independent of weight. | 18F-FDG PET/CT activity; MRI fat fraction; circulating FGF21, NRG4. | β3-agonists (mirabegron), SGLT2 inhibitors, cold exposure, BAT activation/gene therapy.  QSOX1 inhibition; preservation of beige identity (PRDM16 axis) |

Abbreviations: TNF-α, tumor necrosis factor-alpha; IL-6, interleukin-6; NO, nitric oxide; RAAS, renin–angiotensin–aldosterone system; METS-VF, Metabolic Score for Visceral Fat; VAI, Visceral Adiposity Index; VAT, visceral adipose tissue; SAT, subcutaneous adipose tissue; PDFF, proton density fat fraction; PET, positron emission tomography; PPARγ, peroxisome proliferator-activated receptor gamma; BMAT, bone marrow adipose tissue; PVAT, perivascular adipose tissue; BAT, brown adipose tissue; GLP-1RA, glucagon-like peptide-1 receptor agonist; SGLT2, sodium–glucose cotransporter 2; miRNA, microRNA.
